# Linkage of Presumptive Multidrug Resistant Tuberculosis (MDR-TB) Patients to Diagnostic and Treatment Services in Cambodia

**DOI:** 10.1371/journal.pone.0059903

**Published:** 2013-04-25

**Authors:** Sokhan Khann, Eang Tan Mao, Yadav Prasad Rajendra, Srinath Satyanarayana, Sharath Burugina Nagaraja, Ajay M. V. Kumar

**Affiliations:** 1 Stop TB Department, World Health Organization (WHO), Phnom Penh, Cambodia; 2 National Tuberculosis Control Program, Ministry of Health, Phnom Penh, Cambodia; 3 South-East Asia Office, International Union against Tuberculosis and Lung Disease (The Union), New Delhi, India; 4 World Health Organization (WHO), New Delhi, India; Institut de Pharmacologie et de Biologie Structurale, France

## Abstract

**Setting:**

National Tuberculosis Programme, Cambodia.

**Objective:**

In a cohort of TB patients, to ascertain the proportion of patients who fulfil the criteria for presumptive MDR-TB, assess whether they underwent investigation for MDR-TB, and the results of the culture and drug susceptibility testing (DST).

**Methods:**

A cross sectional record review of TB patients registered for treatment between July-December 2011.

**Results:**

Of 19,236 TB patients registered, 409 (2%) fulfilled the criteria of presumptive MDR-TB; of these, 187 (46%) were examined for culture. This proportion was higher among relapse, failure, return after default (RAD) and non-converters at 3 months of new smear positive TB patients (>60%) as compared to non-converters at 2 months of new TB cases (<20%). Nearly two thirds (n = 113) of the samples were culture positive; of these, three-fourth (n = 85) grew Mycobacterium tuberculosis complex (MTBc) and one-fourth (n = 28) grew non-tuberculous Mycobacteria. DST results were available for 96% of the MTBc isolates. Overall, 21 patients were diagnosed as MDR-TB (all diagnosed among retreatment TB cases and none from non-converters) and all of them were initiated on MDR-TB treatment.

**Conclusion:**

There is a need to strengthen mechanisms for linking patients with presumptive MDR-TB to culture centers. The policy of testing non-converters for culture and DST needs to be reviewed.

## Introduction

Globally, of the estimated 290,000 cases of multidrug resistant tuberculosis (MDR-TB) among the notified pulmonary TB cases in 2010, just over 50,000 cases (18%) were diagnosed and among those only 45,553 (16%) were enrolled on MDR-TB treatment under the National Tuberculosis Programmes (NTP) [1]. Detection of MDR-TB requires *Mycobacterium Tuberculosis* culture and drug susceptibility testing (DST) facilities. Due to resource constraints, in many high TB burden countries, only a few laboratories (located at tertiary care health centres) offer such services [2]. The journey begins with identification of presumptive MDR-TB patients in the peripheral health facilities. After identification, patients are either referred or their sputum samples collected and transported to the designated culture and DST facilities and those diagnosed to be MDR-TB are initiated on treatment. Several patients may be lost along the way in this journey due to operational challenges: eligible patients may not be identified, many are not referred and/or may not have their sputum collected and transported to undergo culture and DST, and of those diagnosed to be MDR-TB, many are not initiated on treatment as has been shown in a recent publication from India [3].

Cambodia with a population of 14 million is one of the 22 high TB burden countries in the world; around 61,000 people were estimated to fall ill from TB each year including around 25,000 infectious sputum smear positive cases [1]. According to the most recent drug resistance survey in Cambodia (2006), the prevalence of MDR-TB among new and previously treated cases was 1.4% and 11% respectively. This translates to about 490 MDR-TB cases every year [1].

In 2007, NTP in Cambodia initiated the Programmatic Management of Drug-Resistant TB (PMDT) services based on World Health Organization (WHO) guidelines to treat MDR-TB patients [4] through three separate pilot projects and scaled up to cover the entire country in 2011.According to the programme guidelines, the operational districts (ODs) are required to identify patients with presumptive MDR-TB, collect and transport their sputum samples to any of the three designated referral laboratories for *Mycobacterium* culture and when the culture is positive, the culture samples are sent to the National Referral Laboratory (NRL) for DST.

The NRL routinely reports the results of sputum samples tested to the NTP. This information, though useful for individual patient management, is insufficient from a programme perspective, to assess the efficiency of the programmatic processes in identifying all eligible presumptive MDR-TB and linking them to PMDT services. An operational research study was therefore undertaken to assess i) the number (proportion)of patients who fulfil the criteria of presumptive MDR-TB and their characteristics, ii) whether their sputum samples were examined for culture and DST, iii) the number (proportion) diagnosed as MDR-TB and their resistance patterns, and iv) whether they were initiated on MDR-TB treatment regimen.

## Methods

### Study Designs

This is a cross-sectional descriptive study which involved review of records routinely maintained under NTP.

### Settings

Cambodia has 24 provincial health departments (PHD) and 8 National Hospitals (NH) which covers 77 ODs and more than 1000 health centres (HC) and health posts (HP). The tuberculosis case definitions and management is as per the WHO TB treatment guidelines [5].

### Definition of Presumptive MDR-TB Under the Cambodia NTP

The following group of TB patients fulfil the criteria of presumptive MDR-TB: smear positive previously treated patients who define as relapse, return after default (RAD), and failure; new smear positive pulmonary TB patients who sputum remains smear positive at month 2 or 3 of treatment; symptomatic close contacts of known MDR-TB patient, and new smear positive with Human Immunodeficiency Virus (HIV) infected patients. All programme staff in the districts have been trained to identify patients with presumptive MDR-TB and refer them or collect and transport their sputum samples to one of the three designated referral laboratories which have the capacity to perform culture and species identification. One of them, which is also the National Reference Laboratory (NRL) has the capacity to perform DST as well. The sputum samples are cultured by liquid culture using MGIT 960 with a turnaround time ranging from 4–8 weeks. Those who are found to be culture positive for Mycobacterium tuberculosis are tested for DST at the NRL. Resistance to at least isoniazid and rifampicin was defined as MDR-TB, as per the WHO definition [6]. The confirmed MDR-TB patients are traced back in the field and initiated on MDR-TB treatment.

### Study Population

The study population included TB patients over age 15 years who fulfilled the criteria of presumptive MDR-TB among all TB patients registered for treatment during the period July to December 2011in all the 77ODs and 5 National Hospitals (NH). These patients were identified in the TB registers maintained at all the ODs. Symptomatic close contact of known MDR-TB patients and new smear positive HIV-infected patients were excluded from the study as they could not be identified from the TB registers.

### Data Collection and Variables

The study was conducted by the Principal Investigator with support from the other co-investigators and the programme staff. A list of all patients with presumptive MDR-TB was prepared and name of each patient was matched with that in the culture and DST registers at all the three designated referral laboratories. If the name of the presumptive MDR-TB patient in the list was not found in the culture and DST register for the period July 2011 to March 2012, then the patient’s specimens were considered to have not reached the laboratory. To ascertain if confirmed MDR-TB patients were started on treatment, names were matched with MDR-TB treatment (Category IV) register of all MDR-TB treatment sites in the country. The collected variables were included: TB registration number, age, sex, type of TB, whether sputum samples were examined for culture/DST, culture result, DST result, and treatment initiation status. Unfortunately the information on HIV status was not collected. Even though, base on the 2011 annual report of NTP showed that HIV infected among TB patients was less than 1%. The collected data was double entered, validated and analyzed using Epi Data.

### Ethics Approval

The study protocol was reviewed and approved by the National Ethic Committee for Human Research of Cambodia, the Ethics Advisory Group of the Union, and the Ethics Review Committee of WHO Western Pacific Regional Office. Informed consent was waived by the ethics committee as study did not involve direct interaction with the patient. The researchers did not record the patient's name. A unique ID (OD name+ID of OD TB register) was assigned to each record after completing the data collection. The file that contained both the patient name and ID was secured in a place that was only accessible to the principal investigator, while the file that included the ID only was shared.

## Results

**Figure 1:**
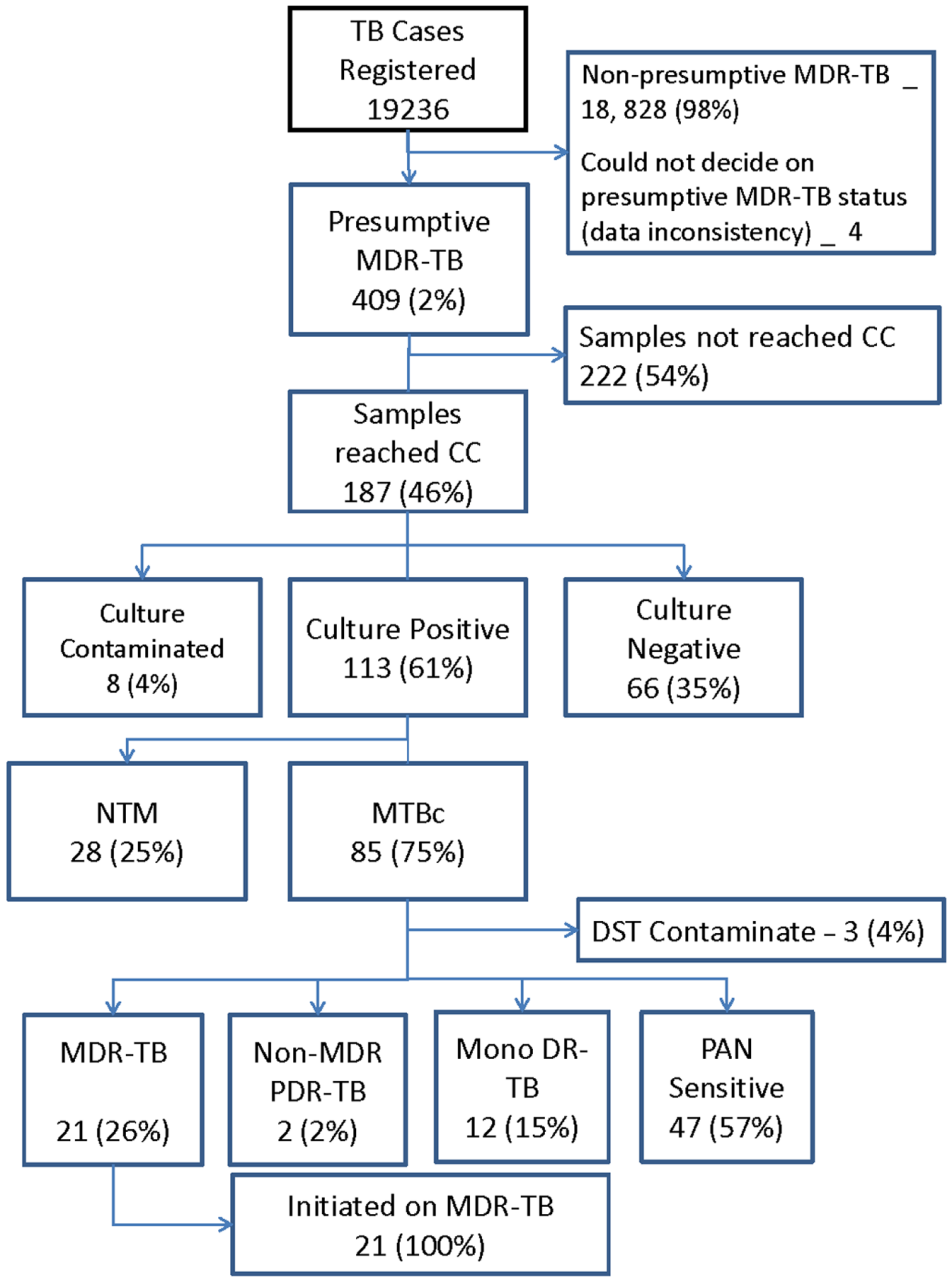
Status of linking presumptive MDR-TB patients to diagnostic and MDR-TB treatment services in Cambodia (2011). CC = Culture center, MTBc = Mycobacterium Tuberculosis complex, NTM = Non tuberculosis mycobacteria, DST = Drug Sensitivity Testing, MDR-TB = Multi drug resistant tuberculosis (resistant at R & H), PDR-TB = Poly drug resistant tuberculosis (resistant more than one but not R & H together), MonoDR-TB = Mono drug resistant tuberculosis.

The study population and the proportion linked to PMDT services are described in **Figure 1**. Of 19,236 TB cases registered in the country during the second half of 2011, 409 (2%) fulfilled the criteria for presumptive MDR-TB and 187 (46%) of these patients had their sputum samples examined for culture. Nearly two thirds of these samples tested were culture positive. One fourth of those tested culture positive were non-tuberculous mycobacteria. Overall, 21 patients were diagnosed as MDR-TB and all of them were initiated on MDR-TB treatment.

The characteristics of patients with presumptive MDR-TB are given in **Table 1**. Nearly two-thirds of the presumptive MDR-TB patients were male and median age was 48. About 46% of them were relapse TB cases and another 40% were 2-month non-converters among new smear positive TB patients. Nearly 70% of relapse, failure, RAD, and non converter at 3 months cases had their specimens examined for culture and DST as compared to only 16% among the non-converters at 2 months.

**Table 1:** Characteristics of presumptive MDR-TB patients and their status throughout the MDR-TB diagnostic pathway, Cambodia, 2011.

	A) Number with presumptive MDR-TBs	B) Of A, samples were received at culture center	C) Of B, culture positive for Mycobacterium	D) Of C, culture positive were MTBc	E) Of C, culture positive were NTM	F) Of D, DST result available	G) Of F, confirmed MDR-TB
	N	N (%)	N (%)	N (%)	N (%)	N (%)	N (%)
Total	409	187 (46)	113 (60)	85 (75)	28 (25)	82 (96)	21 (26)
Type of MDR-TB Suspects							
Relapse	187	119 (64)	80 (67)	64 (80)	16 (20)	63 (98)	13 (21)
Failure	25	17 (68)	11 (65)	9 (82)	2 (18)	8 (89)	8 (100)
Treatment after default	6	4 (67)	4 (100)	4 (100)	0	3 (75)	0
Non-converter at 2 months^1^	163	26 (16)	12 (46)	5 (42)	7 (58)	5 (100)	0
Non-converter at 3 months^2^	28	21 (75)	6 (29)	3 (50)	3 (50)	3 (100)	0

**Note:**

MTBc = Mycobacterium tuberculosis complex, NTM = Non tuberculosis mycobacterium, MTB-TB = Multi drug resistant tuberculosis, DST = Drug Susceptibility Test, 1 New smear positive patient whose sputum remained smear positive at 2 months, 2 New smear positive patient whose sputum remained smear positive at 3 months.

The drug resistance patterns in relation to the different categories of presumptive MDR-TB are given in **Table 2**. Overall, 21 cases of MDR-TB were diagnosed and placed on MDR-TB treatment. All the MDR-TB cases were diagnosed among relapse and failure TB patients. Those cases that had mono rifampicin resistance were also placed on MDR-TB treatment.

**Table 2:** Drug resistance pattern among different types of presumptive MDR-TB, Cambodia, 2011.

	Total with DST result	Resistant Pattern								Pan- Sensitive
		MDR-TB				PDR-TB	Mono DR-TB			
Patient type	N	HRES	HRE	HRS	HR	HS	H	R	S	
Relapse	63	6	2	2	3	2	6	2	4	36
Failure	8	1	3	1	3	0	0	0	0	0
Treatment after default	3	0	0	0	0	0	0	0	0	3
Non-converter at 2 months^1^	5	0	0	0	0	0	0	0	0	5
Non-converter at 3 months^2^	3	0	0	0	0	0	0	0	0	3
Total	82	7	5	3	6	2	6	2	4	47

Note:

**MDR-TB** = Multi drug resistant tuberculosis, **PDR-TB** = Poly drug resistant tuberculosis, **Mono DR-TB** = Mono drug resistant tuberculosis, **H** = Isoniazid, **R** = Rifampicin, **E** = Ethambutol, **S** = Streptomycin.

1 New smear positive TB patient whose sputum remained smear positive at 2 months.

2 New smear positive TB patient whose sputum remained smear positive at 3 months.

## Discussion

This is one of the first studies from Cambodia to study the adequacy and efficiency of linking presumptive MDR-TB patients to PMDT services. The major strength of the study is that the information was gathered from all the operational districts and hence the study findings can be generalized to the whole country. This operations research study identified major issues that have important implications for policy and practice in Cambodia.

First, only 46% of presumptive MDR-TB cases were examined for culture. This indicates an urgent need to strengthen the programmatic processes of identification and linkage of presumptive MDR-TB cases to PMDT services. It is encouraging to note that linkage is much better among relapse, failure, return after default, and non-converter at 3 months of TB cases with more than two-thirds getting examined for culture. Given the resource constraints, relapse, failure, return after default, and non-converter at 3 months of TB cases have been accorded a higher priority by NTP and it is reflected in the performance. The apparent low performance among 2-month non-converters is indicative of lower priority as well as lower levels of drug resistance among them. This reflects operational challenges in implementation of PMDT services. Due to some variables such as knowledge, patient referred. geographic origin, distance from health facilities, income, ethnic or other minorities, and HIV were not collected for the study, so the possible reasons for suboptimal linkage could be - that the peripheral health staffs were not adequately trained to identify all patients with presumptive MDR-TB; that the patients were not able to reach the laboratories due to the cost and effort involved in accessing these services; and/or because of the non-functioning of the sputum collection and transportation mechanism. Studies from China [7] and India have also identified similar system weaknesses [3] where the culture and DST laboratories are not decentralised. The programme should develop innovative strategies for linking patients with presumptive MDR-TB to culture centers. This may involve training of health personnel for early identification of eligible cases, reimbursements for patients to go to sputum culture centres, instituting a mechanism for sputum collection and transportation and/or increase the number of accredited laboratories. Decentralized deployment of rapid molecular diagnostics such as Xpert MTB/RIF can be another way of reducing the attrition in the diagnostic process. It is interesting to note that all the MDR-TB cases came from relapse, failure, and return after default of TB cases. No MDR-TB case was diagnosed among the non-converters. Instead of non-converters, we believe that retreatment others (pulmonary smear negative retreatment TB case) could provide a potential opportunity of diagnosing drug resistance due to the sensitivity of Xpert MTB/RIF is up to 67% among smear negative microscopy [8] and the sensitivity of culture is much higher than Xpert MTB/RIF. We would recommend to NTP to include the pulmonary smear negative retreatment TB case into the criteria of presumptive MDR-TB and future drug resistant prevalent survey.

Second, it was encouraging to notice that all diagnosed MDR-TB patients were initiated on MDR-TB treatment without any further losses to follow-up. This indicates that the programmatic processes adopted to track and treat MDR-TB patients after diagnosis is quite efficient and needs to be sustained. This is excellent and differs from the findings in India, where there was a considerable attrition between diagnosis and treatment. However, the numbers treated are too small when compared to estimated number of MDR-TB cases by WHO. WHO estimates assume that prevalence of MDR-TB is similar among smear positive and smear negative TB cases and this may be resulting in overestimating the burden of MDR-TB, given that there is no information on MDR-TB prevalence among smear negative TB cases. This calls for revisiting the process of estimating MDR-TB burden. Future prevalence surveys should be designed to include smear negative TB patients in the sample.

Third, a large proportion of culture positive samples grew Non-tuberculosis Mycobacterium. This is a concern for the programme to address. It is well known that the anti-tuberculosis treatment regimens used under NTPs are not effective for treating NTM [9]. At the same time, there is limited global guidance available to manage such situations. This necessitates the Cambodian NTP to review their programme policies and provide appropriate guidance to the practitioners in both public and private sector.

### Limitations

First, this research relied on the records maintained routinely under programme settings whose accuracy cannot be ensured at all times. Since the programme is supervised routinely by the programme managers and includes on site visit and periodic cross checking of information, we believe that there are unlikely to be any major deficiencies.

Second, after identification of patients as presumptive MDR-TB, we cross checked the information on whether the patients underwent culture and DST at the three accredited Public health laboratories. If the patients had reached other public health laboratories which are not accredited or had undergone culture and DST at the private laboratories, then we would have missed this information.

Third, the follow-up period we provided to assess whether sputum specimens reached culture laboratory varied among the patients in the cohort. Presumptive MDR-TB cases registered the end of the study period would have had limited window of time to reach the culture laboratory, although we cross checked culture laboratory record for 3 additional months after the last case of 2011 was registered. If the sputum samples of such patients were examined after March 2012, we would have missed capturing information on those cases.

### Conclusions

More than half of presumptive MDR-TB patients were not examined for culture and DST indicating the need to strengthen the programmatic processes involved including decentralized deployment of molecular-based, rapid diagnostic technologies. However, all diagnosed MDR-TB cases were initiated on treatment indicating good linkages between diagnosis and treatment.
